# A cluster-randomized multi-level intervention to increase latrine use and safe disposal of child feces in rural Odisha, India: the Sundara Grama research protocol

**DOI:** 10.1186/s12889-019-6601-z

**Published:** 2019-03-18

**Authors:** Bethany A. Caruso, Gloria D. Sclar, Parimita Routray, Fiona Majorin, Corey Nagel, Thomas Clasen

**Affiliations:** 1 Department of Environmental Health, Rollins School of Public Health, Emory University, Atlanta, GA USA; 2 Independent Consultant, Bhubaneswar, Odisha India; 3 Department of Disease Control, Faculty of Infectious and Tropical Diseases, London School of Hygiene and Tropical Medicine, London, UK; 4 College of Nursing, University of Arkansas for Medical Sciences, Little Rock, AR USA

**Keywords:** Open defecation, Sanitation, Toilet, Behavior change, Social norms, Risk perceptions, Motivations, Multi-level intervention, Theory-based intervention

## Abstract

**Background:**

Despite health benefits of sanitation, an estimated 12% of the global population practices open defecation, including an estimated 50% of the population of India. Current estimates, however, do not include households that own toilets but do not use them, suggesting that the actual number of people defecating in the open is underestimated. This protocol describes a cluster randomized controlled trial to evaluate an intervention specifically designed to increase latrine use, including the safe disposal of child feces, in rural Odisha, India.

**Methods:**

The trial engages 66 villages in Puri district, 33 randomly allocated to receive the intervention and 33 to serve as controls. The primary outcome is latrine use and is recorded at baseline and endline for all members of all households that own latrines in all trial vilalges. Additional data on determinants of latrine use and safe child feces disposal are also collected to assess change based on the intervetntion. A process evaluation assesses the delivery of the intervention and qualiative research takes place in non-trial villages as well as post-endline in trial villages to help explain trial findings.

**Discussion:**

This is one of four trials taking place simultaneously in rural India with latrine use as the primary outcome. All four studies use the same outcome to gerenate comparable data across sites that can serve the government of India. The trial in Odisha is unique in that it collects latrine use data from all potential users in all households that own latrines, enabling a thorough view of the sanitation situation and factors that influence use at the community level. That latrine use is collected via self-report is a limitation, however any bias in reporting should be the same across villages and not impact the overall assessment of intervention impact.

**Trial registration:**

This trial is registered at clinicaltrials.gov: NCT03274245.

## Background

Sanitation has been found to be protective against diarrheal disease, active trachoma, schistosomiasis, some soil-transmitted helminth infections and stunting [1]. Despite these health benefits, an estimated 12% of the world’s population (892 million people) practices open defecation [2]. Current estimates, however, do not include households that own toilets but do not use them, suggesting that the actual number of people defecating in the open is underestimated. A systematic review by Garn et al. [3] found that increases in sanitaiton coverage did not equate to use. With a post hoc regression analysis, the authors found latrine use only increased 5.8% for every 10% increase in latrine coverage [3].

In India, the 2011 national census found that 49.8% of the population defecated in the open, to which the government excelerated focus on sanitation by responding with a series of sanitation campaigns intended to increase sanitation coverage [4, 5]. The current version of the national sanitation program, known as Swachh Bharat Mission (SBM), was initiated under Prime Minister Narendra Modi on October 2, 2014 with the goal of making India defecation free by October 2, 2019 [6]. As of December 3, 2018, the SBM claims to have constructed 89,369,285 latrines amounting to approximately 97% coverage. As of March 7, 2019, the SBM claims to have constructed 92,358,614 latrines amounting to approximately 98.9% coverage [7]. However, use of household latrines has been a consistent challenge. In research across five Indian states with 3235 households and 22,787 respondents, Coffey et al. [8] found that over 40% of households with working latrines had at least one family member still practicing open defecation [8]. They also found that people were more likely to use privately built latrines than those built with government funds, regardless of their wealth [8]. A follow-up study found only 44% of rural inhabitants from these states to be defecting in the open compared to 70% in 2014; however 23% of latrine owners still practice open defecation in 2018, which is unchanged from 2014 [9]. In an assessment of latrines constructed in villages in Odisha under the Total Sanitation Campaign (TSC), a campaign that preceeded the SBM, Barnard et al. [8, 10] found that 39% of latrines were not in use by any household member. The lack of sanitaiton use has been cited as a potential explanation as to why large-scale community level sanitation interventions have failed to lead to health benefits [11, 12]. These results have led to a call to invest in the development and assessment of interventions that specifically promote and evaluate defecation behaviours above and beyond accelerating access alone [13].

There is a compelling need to identify strategies that increase and sustain the practice of latrine use. However, research in India also suggests that such strategies must address multiple factors associated with continued open defecation. Studies have identified a variety of factors related to why members of rural Indian households elect to defecate outside rather than use a latrine: incomplete or poor quality latrines, lack of water access in latrines, concern for latrine pits filling too quickly, large family size, sex and age of user, livelihood type, remoteness, ability to socialize and roam when going for open defecation, general preference for open defecation, and a lack of habit for latrine use [9, 14–18]. This broad range of conditions illustrates the complexity of the challenge of ending open defecation.

There is also a need to increase the practice of safe disposal of child feces. Households that have access to improved sanitation often do not always dispose of their children’s feces into the latrine [19–24]. Instead, child feces are disposed of in the household’s solid waste pile or other open location, enabling potential exposure to harmful fecal pathogens and increasing likelihood of negative health impacts [25]. The latest Indian National Family Health Survey (NFHS) found that the faeces of only 35% of children under age five ended up in a latrine (22% from the child defecating directly into a latrine, and 13% from subsequent disposal into a latrine) [26]. Evaluations of Indian sanitation campaigns have found limited impacts on child faeces disposal practices. In an assessment in Odisha, India of villages covered by the Total Sanitation Campaign (TSC), which aimed to reduce open defecation primarily through latrine construction, safe disposal of child faeces increased from 1.1% at baseline to 10.4% in intervention households compared to 3.1% in control households (RR: 3.34; 95% CI: 1.99–5.59) [19]. While some improvements in child faeces disposal were achieved, the majority of faeces still ended up in the environment. It’s important to note that the TSC intervention included sparse behaviour change messaging to increase latrine use, including use by children, or for safe disposal of child faeces.

### Objectives

As part of a larger program of research improving latrine use in India [27], we sought to develop and assess the effectiveness of a low-cost behavior change intervention that could be deployed at scale to improve latrine use and the safe disposal of child feces in Odisha. The intervention is based on formative research and behavioral theory. This paper describes the design of a two arm (parallel group design), cluster randomized controlled trial (CRT) to evaluate this intervention.

Our primary research question is: Is the intervention effective in improving latrine use and safe disposal of child feces among households in rural communities in Odisha that received latrines under the SBM or prior sanitation campaigns?

We also sought to address the following secondary research questions: (i) Does the intervention compel households that do not own latrines to construct a latrine?; (ii) Does the intervention influence the identified determinants of behaviour? Specifically, are behavioral determinant scores (i.e. scores for social norms, abilities, physical opportunity, risk perception, motivation, and self-regulation) higher at endline among latrine owners in intervention villages compared to latrine owners in control villages?; and (iii) Are behavioral determinant scores (i.e. scores for social norms, abilities, physical opportunity, risk perception, motivation, and self-regulation) associated with latrine use?

This protocol follows the SPIRIT guidelines; the associated WHO checklist is also completed (See Supplemental Files).

## Methods

### Design

The intervention is designed to be delivered at the village level, thus we elected to employ a CRT trial study design, which also allows us to include village-level systems and dynamics, including feedback loops and spillover effects [28]. Clusters are rural villages in Puri district, Odisha, India. Sample size calcultions concluded that we required 66 villages in order to assess effects on the primary outcome; an additional 6 villages were included in the study for qualitative reseach. In all 66 trial villages we censused all households and consented and baselined the latrine-owning households. Thereafter, we randomly assigned half of the villages to receive the intervention while the balance served as controls. We conducted a process evaluation to assess intervention delievery across the 33 intervetnion villages.

**Fig. 1:**
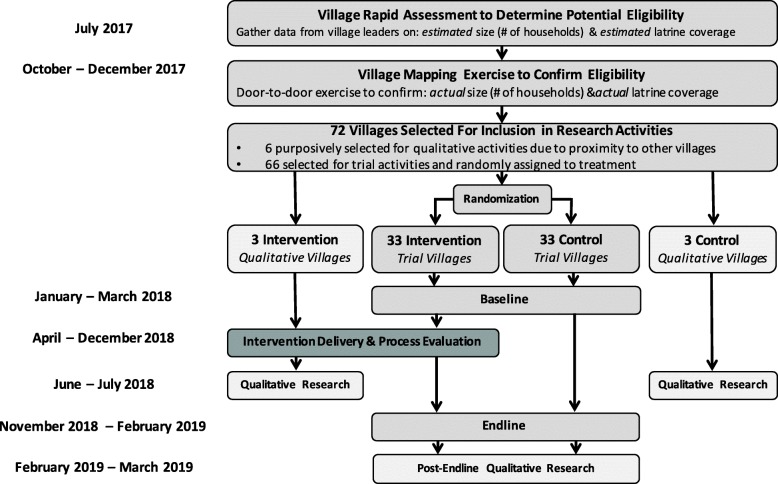
Flow Diagram for Cluster Randomised Trial to Assess Impact of *Sundara Grama* Intervention on Latrine Use in Rural Odisha, India

Qualiative research is used to understand the intervention from participant perspectives and explain trial findings. We are conducting qualitative research in an additional subset of six non-trial villages, three of which received the intervention at the same time as those involved in the trial. Qualitative research in intervention villages seeks to understand village members’ ‘satisfaction’ with the intervention, including content and delivery, as part of the process evaluation. Qualitative research in non-intervetnion villages seeks to identify spillover effects. Qualitative research will also be conducted in trial villages after endline data collection to assist interpretation of quantitative findings. See Fig. 1 for overall research design.

### Setting and study population

The study is taking place in Delang, Nimapada, and Pipili blocks in rural Puri district, Odisha, India. In puri, 84% of the population resides in rural areas and 23% of rural women are illiterate [5]. According to the 2015–2016 Indian NFHS, 37% of rural households in Puri district have an improved sanitation facility – the same percentage of those living in rural areas at the national level albeit somewhat higher than the overall state level (23% in Odisha).

Puri was previously the site of a cluster-randomized trial that sought to understand the impact of the Indian Government’s TSC on various indicators of child health, including diarrhea, stunting, and parisitic infection [29]. The study found no impacts on health and low rates of latrine coverage and use were posited as potential reasons [11].

The percentage of households practicing safe child feces disposal in Odisha is the lowest in the country, with only 12.5% of feces safely disposed [26]. Among rural households in the State of Odisha in villages where the TSC had been implemented at least 3 years prior, a cross-sectional study found that 81.4% of child feces were reported to be disposed of unsafely, with the majority of feces being deposited with the household’s solid waste [20].

### Inclusion/ exclusion

To be eligible for inclusion in the study, we sought villages that had not been declared open defecation free by the Government of India, had 50–150 households, and minimum 60% latrine coverage. These criteria reflect both the study needs (coverage and household size influence sample size estimates) and the limits of our funding (larger villages would require more inputs and thus more costs). Because latrine construction remains ongoing across the district as a result of the SBM, no accurate and up-to-date sampling frames existed with the information needed for village selection, namely village size and latrine coverage. To identify potentially eligible villages, we met with village leaders in the identified blocks to create a preliminary sampling frame of villages that could be of the appropriate size and coverage if confirmed. We then visited the potentially elible villages to conduct a census of all households and record whether each owned a latrine to determine coverage. As it was difficult to find villages that satisfied our a priori eligibility criteria of 50–150 households and minimum 60% latrine coverage, we allowed villages outside the size range and below the minimum coverage provided that we maintained adequate statistical power.

If two or more villages are immediately adjacent to each other, we only selected one so as to minimize the likelihood of spillover. While villages that have been declared by the Indian Government as ‘Open Defecation Free’(ODF) prior to baseline were ineligible, villages that are declared ODF after baseline activities have been completed will remain enrolled in the study.

We only engage participants over age 18 in data collection activities.

### Intervention

Formative research was undertaken in January–April 2017 to design a theoretically-informed sanitation behavior change intervention. As per guidance from the study funder, the International Initaitive for Impact Evaluation (3ie), the the intervention should aim to increase latrine use among latrine owning households at a cost of no more than 20USD per household on average.

A local NGO, Rural Welfare Institute (RWI), delivered the intervention package, called *Sundara Grama* or *beautiful village*. The intervention includes activities at the vilage and household levels, with an additional activity for mothers with children under age five. Trained community mobilizers, hired and managed by RWI, organized and facilitated the intervention activities in each treatment village. For households with non-functional latrines, masons were hired to provide limited repairs as was feasible in terms of timing and cost.

The intervention included the following village-level activities:

- Pre-intervention community visits: Before the start of intervention activities, community mobilizers made preliminary visits to each village to build rapport with key village stakeholders, foster support for the intervention, plan intervention logistics (e.g. location and date for activities), and learn about the social dynamics of each village.
- Palla performance: The first activity to take place in each village was a traditional folk art performance known as a ‘palla,’ which was performed by local troops hired and trained and managed by RWI. They included songs and skits to provide action messaging about the health and non-health benefits (i.e. comfort, privacy) of latrine use and safe disposal of child feces.
- ‘Colored powder transect walk’: Following the palla performance, community mobilizers conducted a surprise transect walk of the village, inviting village members to participate. Community mobilizers distributed bags of brightly colored powder to encourage participants to mark piles of feces that they saw during the walk. The aim of the walk was to have village members reevaluate their environment and vividly recognize the amount of fecal contimation in their village due to open defecation.
- Community meetings: Community meetings, one for women and one for men facilitated participants to decide upon a set of action steps to achieve the goal of a ‘clean, healthy, and beautiful village’ including latrine use by all members of the household at all times. Participants were also asked to identify ‘positive deviant’ households, households where all the members always used their latrine for defecation for latter intervention activities.
- Positive deviant household recognition: Households identified as positive deviants were provided a banner to display in front of their house to publically recognize and praise their contribution to achieving a ‘clean, healthy, and beautiful village.’
- Village wall painting: For the final village level activity, artisans painted a mural that shows a map of all of the households in the village and distinctly identified the positive deviant households. It serves as a reminder to motivate households to use their latrines.

The intervention also included a group level activity:

- Mothers meetings: Community mobilizers held meetings open to all mothers/caregivers of children under age five to provide the necessary action knowledge and hardware (scoops, potties) for mothers/caregivers to perform safe child feces disposal.

Finally, the following household-level activities targeted all latrine-owning households:

- Household visits: A community mobilizer made individualized visits to each household with a latrine. The aim of the visit was to reflect on the intervention activities to date, reiterate key messages, and have household members pledge their commitment to the village goal of a ‘clean, healthy, and beautiful village’, which includes latrine use by all members of the household at all times. The household was given a poster to serve as a reminder of their commitment.
- Latrine assessment and repairs: Lastly, identified households received minor repairs to their latrine from masons so that a key barrier to use—lack of access to a *functional* latrine— was removed.

There is no plan to provide control villages with the intervention at the end of the trial.

### Study outcomes and measures

#### Primary outcome

The primary outcome is *latrine use*, including use for the safe disposal of child feces. Latrine use is assessed for every member of all households that own a latrine in engaged study villages at both baseline and endline. At baseline and endline, the survey respondent is first prompted to list all household members that reside in the household. Then, starting with the respondent, the following is asked for every household member noted in the household roster: “The last time [NAME] defecated, did [NAME] defecate in the open or use the latrine?” Response options include: Open, Latrine, Somewhere else (i.e. potty, nappy, bedpan). If the household member is present and able, the member provides a response. If not, the survey respondent answers on their behalf. If ‘open’ or ‘somewhere else’ is indicated, the respondent is asked follow-up questions to discern if the feces were eventually safely disposed into a latrine. We acknowledge that self-report is not ideal and reporting bias is plausible, but we are asking for ‘last defecation event’ over ‘usual’ behavior, which has been demonstrated to be more robust [30]. This series of questions to determine latrine use and safe disposal of feces was agreed upon by all four research teams funded by 3ie to enable comparison of study results [27].

#### Secondary outcomes

Latrine coverage is assessed at baseline and endline by asking all households if they have a latrine. If they have a latrine, the household respondent is asked to participate in a more extensive survey that includes latrine observations to verify presence, condition, and signs of use.

Determinants of latrine use and child feces disposal were identified during the formative research phase and include: Risk Perceptions, Ability, Social Norms, Motivation, Physical Opportunity, and Self-Regulation. Our survey includes several indicators to represent each of these latent constructs. Baseline and endline scores will be compared to assess change attributable to the intervetntion.

### Sample size

#### Trial

The sample size for the trial was based on the primary outcome of reported latrine use during the last defecation event. We used a simulation-based approach to account for the inclusion of a baseline measure of latrine use in statistical models and to adjust for within-person and within-cluster correlation [31]. Sample size estimates were also checked using the *clustersampsi* add-on package in Stata v.14 (StataCorp, College Station, Texas, USA) [32]. The simulation parameters were derived from data on latrine use collected from 2011 to 2013 during a large-scale sanitation trial in Odisha [11]. Based on those data, we assumed a baseline prevalence of latrine use of 45%, a village-level ICC of 0.10, and a within-person correlation from baseline to follow-up of 0.60. Estimates of household latrine coverage and village size were generated from a rapid assessment of villages in the study area conducted in 2017. No previous trials have investigated the effect of interventions specifically aimed at increasing latrine use to inform sample size estimates. We selected a 10% increase in absolute prevalence of use (from 47 to 57%) as a reasonable minimum intervention effect assuming that the intervention will have a greater effect than increases in coverages have demonstrated (see Garn 2017 who found an increase 5.8% for every 10% increase in latrine coverage in post hoc regression analysis). Assuming an average of 292 eligible participants per village (cluster size coefficient of variation = 0.35), 10% loss to follow-up, 80% power, and 0.05 significance level, we calculated a required sample size of 33 villages per arm.

We will also randomly select a subset of 20 households per trial village to answer questions on determinants to latrine use behaviours. We are aiming for 10 women and 10 men per village as available at both baseline and endline. With 66 villages and 20 individuals per household, we aim to engage a total of 1320 individuals (660 women and 660 men).

Further, all caregivers of children under age five who own a latrine will be asked about child feces disposal knowledge, practices, and behavioural determinants. From our previous work, we anticipate that there will be approximately 10 households per village (average size 100) that will have at least 1 child under age five. As such, we anticipate engaging 660 caregivers of children under age five across the 66 trial villages at both baseline and endline.

#### Qualiative research

We selected six additional non-trial villages for qualitative research. We anticipate engaging approximately 110 individuals in these villages over the course of the study via in-depth intervention (IDIs) (~ 30) and focus group discussions (FGDs) (~ 8 FGDs with 6–10 participants each).

Finally, after endline data collection, we will carry out IDIs with individuals in trial intervention villages that initiated latrine use by endline compared to baseline, with individuals who did not change behaviour at all despite having a latrine, and with individuals who did not have a latrine at baseline but had one by endline. We will also carry out FGDs with men and women in intervention villages that saw the greatest overall change in latrine use and the least overall use to determine what factors contributed to change and if and how the intervention components influenced that change. Sample size numbers are not final at this stage, though we anticipate approximately 30 IDIs and 8 FGDs with approximately 6–10 participants in each.

### Recruitment

Discussions with village leaders were held during the initial mapping phase to assess willingness to participate. Individual households are visited directly at both baseline and endline to participate in data collection activities.

### Selection of communities and randomization

Following baseline data collection, we randomly allocated villages to control and intervention arms in equal proportion, using stratified randomization to ensure balance on village size and latrine coverage. The 33 villages in the intervention arm are receiving the *Sundara Grama program,* while villages in the control arm will receive no program activities. Village allocation was performed by a study investigator using a computer-generated randomization sequence generated in Stata v.14. Due to the nature of the intervention, neither participants nor study investigators will be blinded to treatment assignment.

We used the village lists generated during the mapping exercise and a random number generator in excel to select the latrine-owning households per trial village to answer questions on determinants to latrine use behaviours. We generated a list of 30 households to be sure we captred at least 20 per village to participate. We then used random number generator in excel to determine if a female or male respondent in each household would be targeted to answer the survey segment.

The additional six non-trial villages engaged in qualitative work alone were purposively selected to include villages that exist in close proximity to trial villages to enable investigation of spillover effects as appropriate.

### Data collection

We hired 13 enumerators and 2 supervisors to carry out baseline and endline data collection. Six enumerators and both supervisors had been a part of previous Emory study teams in Odisha with 2 to 6 years of experience working on sanitation studies. We conducted two training sessions with the survey team prior to baseline (a four-day training in November and a four-day refresher training in January), and will conduct additional training prior to endline. All survey team members are from rural Puri and provided critical feedback on all tools during the training pertaining to the phrasing of questions, Odia translation, necessary skip patterns, and other relevant logistics regarding data collection.

Data are collected using smartphones programmed with open data kit (ODK) [33]. At baseline, all households in each village will be asked if they have a latrine. If they do not, the engagement ends. If they do, a survey will be verbally administered to all consenting households, including questions about latrine use for all family members, at baseline and endline. Observations of latrine facilities will also take place at baseline and endline. At endline, the same procedure will be followed. No plans are in place for retention of households that participated at baseline so as to not be coercive. Latrine ownership will be noted for all households whether they participate in the baseline and endline surveys or not.

Survey questions to understand determinants of latrine use behaviors will only be asked of the household if randomly selected a priori. While we will aim to have 10 women and 10 men per village, men may be hard to find thus we will ask a female to participate from the randomly identified household if a male household member is not available to ensure we have at least 20 participants per village.

Enumerators will ask participants in all latrine-owning households with children under age five to have a primary caregiver answer questions about child feces disposal knowledge, practices, and determinants.

If different participants are identified to complete the behavioral determinants or child feces portions than those initially engaged, they will be consented before data collection.

### Data management

Every Android phone used for quantititve data collection will be secured with a different 4-digit PIN. The phones will be programmed to ensure that the enumerators will be unable to change any settings in the ODK Collect application. Rigorously tested skip patterns and requirements to not skip questions without actively indicating a refusal to answer serve to ensure data quality and minimize missing data. The server used to compile the data will be secured with a 20-character password. The data will be pulled from the server and pushed to a Box folder, secured with a different 20-character password. A limited number of data collection activities as part of the process evaluation will utilize paper surveys. These will be secured in a locked office and later double entered in excel.

Qualitative activities will be digitally recorded and directly translated into English. All participants will be de-identified during transcription to ensure confidentiality. Participants will be given a code number that will link them to a limited amount of demographic information (age, number of children, etc.) collected on paper surveys and double-entered in excel. All transcribed documents will require a code to access.

All data will only be accessible to Research Assistants and Emory Research faculty, staff, and students named on the IRB to protect confidentiality. Data will be made publically available after analysis of endline data in 2019.

### Analysis of trial data

#### Quantitative analysis

We will conduct an intent-to-treat analysis of differences in the specified outcomes between the treatment and control groups following data collection after the delivery of the intervention. For the primary outcome, we will fit a log-binomial model to yield the prevalence ratio of post-intervention latrine use in persons receiving the intervention relative to controls, adjusting for baseline latrine use and any variables observed to be substantially imbalanced between groups at baseline. We will employ generalized estimating equations (GEE) with an independent correlation structure and robust standard errors to account for village and person-level clustering. The effect of the intervention on secondary outcomes will be assessed using GEE models with the distribution and link functions appropriate to the specific variable. The results of both unadjusted and adjusted models will be reported for all outcomes.

#### Qualitative analysis

Transcripts from qualitative activities will be analysed using thematic content analysis. Members of the research team will begin analysis by reading through transcripts and writing memos about the issues discussed in the location where they collected the data. The memos will inform the creation of a preliminary codebook. The preliminary codebook will be shared among members of the research team and refined. Researchers will then use the final codebook to apply codes to the data collected. Once coding is complete, researchers will write thematic memos. Some memo topics will be pre-determined (deductive). Other memos will be created that are not anticipated based on what is learned from the data collected (inductive).

### Reporting harms, auditing, and dissemination plans

The research team has weekly calls to discuss any issues, including potential adverse events, so action can be taken as needed. A process evaluation field team was present in villages during most intervention activities and can report potential adverse events to the broader research team members. No harm is anticipated and therefore no plans are in place for post-trial care. No plan is in place for auditing the trial.

Research findings will be submitted to peer-reviewed journals for open access publication and presented at conferences. We do not intend to use professional writers. Findings will also be shared with government stakeholders at the local and national levels to enable evidence-based decision making regarding latrine use initaitives. We plan to hold meetings in each participating village in the spring of 2019 to share overall findings.

## Discussion

This study evaluates the impact of a multi-level intervention that aims to increase latrine use by all, including safe disposal of child feces, in households that own latrines in rural Odisha, India. This is one of four studies being rolled out concurrently to evaluate interventions designed to increase latrine use in rural India at an average cost of 20USD per household, and the only of that group to include a specific intervention component for safe child feces disposal. Odisha has the second lowest rate of latrine coverage in India despite SBM efforts [7], representing a critical sanitation challenge. Evidence generated will provide the government with insights to inform programs to encourage latrine use at scale across the state of Odisha, and potentially in other states. Even if the country reaches its goal of becoming open defecation free by October 2, 2019, these findings can also inform government actions to make sure behavior is sustained.

We recognize that there are limitations to how latrine use or non-use, the primary outcome of the study, is collected. While the accuracy of the data may be questioned since it is self-reported or reported by a family member, we expect inaccuracies to be consistent across all households in all study villages and therefore not impact findings. Additionally, this strategy is consistent with the other three studies being undertaken enabling comparison across all study locations.

This is the only evaluation of the four to collect latrine condition and latrine use data from all members of latrine-owning households in each engaged community. This substantial dataset enables nuanced assessment of changes in latrine conditions over time and the influence of latrine conditions on behavior. It also enables the assessment of village-level effects, like village latrine coverage and proportion of village members of different castes, on use. Most importantly, the date generated will provide a comprehensive appraisal of both the current sanitation environment and local practices in the engaged villages, representing a vital resource for local government officials to target sanitation efforts and decision making.

## Abbreviations

CRT: Cluster randomized controlled trial
FGD: Focus group discussion
GEE: Generalized estimating eqs.
IDI: In depth interview
ODF: Open defecation free
SBM: Swachh Bharat Mission
TSC: Total Sanitaiton Campaign

## References

[1] Freeman MC, Garn JV, Sclar GD, Boisson S, Medlicott K, Alexander KT (2017). The impact of sanitation on infectious disease and nutritional status: a systematic review and meta-analysis. Int J Hyg Environ Health.

[2] JMP. Progress on drinking water, sanitation and hygiene: 2017 Update and SDG Baselines. Joint Monitoring Program, World Health Organization, UNICEF; 2017 July 12, 2017.

[3] Garn JV, Sclar GD, Freeman MC, Penakalapati G, Alexander KT, Brooks P (2017). The impact of sanitation interventions on latrine coverage and latrine use: a systematic review and meta-analysis. Int J Hyg Environ Health.

[4] WHO/UNICEF. Progress on drinking water and sanitation: 2015 update and MDG assessment. Geneva; 2015.

[5] Puri District: Census 2011-2019 data. https://www.census2011.co.in/census/district/411-puri.html. Accessed 4 Mar 2019.

[6] Ministry of Drinking Water and Sanitation. Swachh Bharat Mission- Gramin [updated 20 November 2018. Available from: http://swachhbharatmission.gov.in/sbmcms/index.htm.

[7] Ministry of Drinking Water and Sanitation. Swachh Bharat Mission- Gramin Dashboard [updated 3 December 2018. Available from: http://sbm.gov.in/sbmdashboard/Default.aspx.

[8] Coffey D, Gupta A, Hathi P, KhCoffey D, Gupta A, Hathi P, KhCoffey D, Gupta A, Hathi P, KhCoffey D, Gupta A, Hathi P, Khurana N, Spears D, Srivastav N, et al. Revealed preference for open defecation. Econ Polit Wkly. 2014;49(38):43.

[9] Gupta AN, Khalid D, Desphande P, Hathi A, Kapur N, Srivastav S, Vyas D. Spears and D. Coffey. Changes in Open Defecation in Rural North India: 2019;2014-2018

[10] Barnard S, Routray P, Majorin F, Peletz R, Boisson S, Sinha A (2013). Impact of Indian Total sanitation campaign on latrine coverage and use: a cross-sectional study in Orissa three years following programme implementation. PLoS One.

[11] Clasen T, Boisson S, Routray P, Torondel B, Bell M, Cumming O (2014). Effectiveness of a rural sanitation programme on diarrhoea, soil-transmitted helminth infection, and child malnutrition in Odisha, India: a cluster-randomised trial. Lancet Glob Health.

[12] Patil SR, Arnold BF, Salvatore AL, Briceno B, Ganguly S, Colford JM (2014). The effect of India's total sanitation campaign on defecation behaviors and child health in rural Madhya Pradesh: a cluster randomized controlled trial.

[13] Luby S (2014). Is targeting access to sanitation enough?. Lancet Glob Health.

[14] Coffey D, Gupta A, Hathi P, Spears D, Srivastav N, Vyas S. The puzzle of widespread open defecation in rural India: evidence from new qualitative and quantitative data: Working Paper; 2015.

[15] Coffey D, Spears D, Vyas S. Switching to sanitation: understanding latrine adoption in a representative panel of rural Indian households. Soc Sci Med. 2017. 10.1016/j.socscimed.2017.07.001 PMC5641475 28715752

[16] Routray P, Schmidt W-P, Boisson S, Clasen T, Jenkins MW (2015). Socio-cultural and behavioural factors constraining latrine adoption in rural coastal Odisha: an exploratory qualitative study. BMC Public Health.

[17] Sinha A, Nagel CL, Schmidt WP, Torondel B, Boisson S, Routray P (2017). Assessing patterns and determinants of latrine use in rural settings: a longitudinal study in Odisha, India. Int J Hyg Environ Health.

[18] O'Reilly K, Dhanju R, Goel A (2017). Exploring “the remote” and “the rural”: open defecation and latrine use in Uttarakhand, India. World Dev.

[19] Freeman MC, Majorin F, Boisson S, Routray P, Torondel B, Clasen T (2016). The impact of a rural sanitation programme on safe disposal of child faeces: a cluster randomised trial in Odisha, India. Trans R Soc Trop Med Hyg.

[20] Majorin F, Freeman MC, Barnard S, Routray P, Boisson S, Clasen T. Child feces disposal practices in rural Orissa: a cross sectional study. PLoS One. 2014;9(2). 10.1371/journal.pone.0089551 PMC3930746 24586864

[21] WSP. Management of Child Feces: Current Disposal Practices. 2015.

[22] Curtis V, Kanki B, Mertens T, Traore E, Diallo I, Tall F (1995). Potties, pits and pipes: explaining hygiene behaviour in Burkina Faso. Soc Sci Med.

[23] Azage M, Haile D (2015). Factors associated with safe child feces disposal practices in Ethiopia: evidence from demographic and health survey. Archives of Public Health.

[24] Miller-Petrie MK, Voigt L, McLennan L, Cairncross S, Jenkins MW (2016). Infant and young child feces management and enabling products for their hygienic collection, transport, and disposal in Cambodia. Am J Trop Med Hyg.

[25] Bawankule R, Singh A, Kumar K, Pedgaonkar S (2017). Disposal of children’s stools and its association with childhood diarrhea in India. BMC Public Health.

[26] International Institute for Population Sciences (IIPS) and ICF. National Family Health Survey (NFHS-4), 2015–16: District Fact Sheet Puri Odisha. Mumbai: IIPS; 2017.

[27] International Initiative for Impact Evaluation (3ie). Promoting Latrine Use in Rural India [Available from: http://3ieimpact.org/our-work/water-sanitation-and-hygiene/promoting-latrine-use-rural-india.

[28] Hayes RJ, Moulton LH. Cluster randomised trials: Chapman and Hall/CRC; 2017.

[29] Clasen T, Boisson S, Routray P, Cumming O, Jenkins M, Ensink JH (2012). The effect of improved rural sanitation on diarrhoea and helminth infection: design of a cluster-randomized trial in Orissa, India. Emerging themes in epidemiology.

[30] Sinha A, Nagel CL, Thomas E, Schmidt WP, Torondel B, Boisson S (2016). Assessing latrine use in rural India: a cross-sectional study comparing reported use and passive latrine use monitors. Am J Trop Med Hyg..

[31] Arnold BF, Hogan DR, Colford JM, Hubbard AE (2011). Simulation methods to estimate design power: an overview for applied research. BMC Med Res Methodol.

[32] Hemming K, Marsh J (2013). A menu-driven facility for sample-size calculations in cluster randomized controlled trials. Stata J.

[33] Hartung C, Anokwa Y, Brunette W, Lerer A, Tseng C, Borriello G. Open data kit: tools to build information services for developing regions. [Available from: http://citeseerx.ist.psu.edu/viewdoc/summary?doi=10.1.1.176.8017.

